# A palette of bright and photostable monomeric fluorescent proteins for bacterial time-lapse imaging

**DOI:** 10.1126/sciadv.ads6201

**Published:** 2025-04-16

**Authors:** Nathan Fraikin, Agathe Couturier, Romain Mercier, Christian Lesterlin

**Affiliations:** Molecular Microbiology and Structural Biochemistry (MMSB), Université Lyon 1, CNRS, Inserm, UMR5086, 69007 Lyon, France.

## Abstract

Fluorescent proteins (FPs) are pivotal for examining protein production, localization, and dynamics in live bacterial cells. However, the use of FPs in time-lapse imaging is frequently constrained by issues such as oligomerization or limited photostability. Here, we report the engineering of novel cyan, green, yellow, and red FPs that exhibit improved photostability and aggregation properties while retaining high in vivo brightness. We first derived superfolder green fluorescent protein into mChartreuse, a brighter, more photostable, and monomeric fluorophore. mChartreuse was further derived into cyan and yellow variants with enhanced photostability and dispersibility. We also report a mutation that eliminates residual oligomerization in red FPs derived from *Discosoma* sp., such as mCherry or mApple. Incorporation of this mutation in mApple among other substitutions yielded mLychee, a bright and photostable monomeric red FP. These novel FPs advance fluorescence time-lapse analysis in bacteria, and their spectral properties match current imaging standards, ensuring seamless integration into existing research workflows.

## Teaser

Bright and photostable monomeric fluorescent proteins enable long-term time-lapse imaging in *E. coli*.

## INTRODUCTION

Fluorescent proteins (FPs) are essential tools in life science, enabling the tracking of protein localization and gene expression at the single-cell level (*1*). Green Fluorescent Protein (GFP) was initially cloned from the jellyfish *Aequorea victoria* and initiated a revolution in the study of protein localization (*2*). However, wild-type *A. victoria* GFP (avGFP) showed several shortcomings since it matured poorly at 37°C and required the use of phototoxic ultraviolet light (400 nm) to be excited (*3*). Mutagenesis of avGFP enabled the discovery of well-folded variants that were excited by blue light (488 nm), such as enhanced GFP (EGFP), GFP mutant 2 (GFPmut2), or superfolder GFP (sfGFP) (*4*–*6*). GFP derivatives also displayed a tendency to dimerize and cause mislocalization of fusion partners, which could be eliminated by disrupting a small hydrophobic dimerization interface with the A206K substitution (*7*). Substitution of residues critical for fluorescence also enabled the engineering of blue, cyan, and yellow variants of GFP, expanding the palette of usable FPs (*8*, *9*).

Subsequently to the engineering of GFP, the discovery of a red FP (RFP) in *Discosoma* sp. paved the way for multicolor analysis. This *Discosoma* sp. RFP (DsRed) was homologous to avGFP but excited by green light (558 nm), which, therefore, enabled simultaneous imaging of GFP and DsRed with minimal spectral overlap (*10*). However, engineering usable derivatives of DsRed was extremely challenging since this RFP matures slowly, oligomerizes as a tight tetramer, and can stochastically mature as a green side product that overlaps with the spectral properties of GFP (*11*). Bevis and Glick (*11*) overcame the slow maturation of DsRed by random mutagenesis, yielding DsRed-Express, which matured 15 times faster than DsRed. A tour-de-force study by the Tsien laboratory (*12*) reported the monomerization of DsRed-Express into a fast-maturing monomeric RFP (mRFP1) through structure-guided disruption of its AB and AC tetramerization interfaces. While mRFP1 was dim and bleached quickly when excited, subsequent studies evolved a palette of DsRed derivatives with a wide range of properties, which were called mFruits (*13*, *14*). Of all mFruits, mCherry became the standard RFP due to its fast maturation, high photostability, and lack of GFP-like side product (*14*).

Over the years, potentially useful FPs were identified in other organisms. For example, an extremely bright-yellow FP was identified in the cephalochordate *Branchiostoma lanceolatum* and engineered to a monomeric GFP called mNeonGreen (*15*). mNeonGreen was twice as bright as EGFP, with similar photostability and maturation kinetics as the latter (*15*), thereby highlighting the potential for bright and exploitable FPs to be found in organisms beyond *Discosoma* sp. or *A. victoria*. Other recently released FPs such as AausFP1 from *Aequorea australis*, mStayGold from *Cyteais uchidae*, or AzaleaB5 from *Montipora monasteriata* show great promise as bright fluorescent probes (*16*–*18*). Novel FPs could also be engineered de novo from a synthetic template as was the case for the mScarlet family (*19*). Further evolution of mScarlet yielded mScarlet3 and mScarlet-I3, the brightest RFPs to date (*20*).

All FPs have different sets of parameters that dictate setups for which they should be used. These include fluorescence spectrum, brightness, maturation rate, photostability, and dispersibility (*21*). The brightness of FPs is measured as the product of its extinction coefficient and quantum yield (QY) and represents the amount of signal that can be obtained from a given FP. However, another critical parameter that influences in vivo brightness is maturation rate, which defines the kinetics by which a folded dark FP is converted into a fluorescent species by cyclization of three amino acids that become its chromophore. Because bacteria tend to be fast-growing organisms, the use of fast-maturing FPs is of utmost importance in these organisms to achieve high brightness, since dark species of slow-maturing FPs would quickly get diluted by fast doublings (*22*).

Dispersibility, or the tendency to form multimeric structure, is a prime property to look out for when using FPs as fusion tags. Nonmonomeric FPs can cause aggregation artifacts when fused to proteins that form multimeric complexes. This tendency for FPs to aggregate is often evaluated using the organized smooth endoplasmic reticulum (OSER) assay, in which the FP of interest is addressed to the cytoplasmic side of the endoplasmic reticulum membrane (*21*, *23*). In this setup, multimeric FPs cause quantifiable morphological abnormalities of the endoplasmic reticulum, thereby enabling in vivo evaluation of FP suitability for use in fusion tags (*23*). However, some FPs that do not affect endoplasmic reticulum morphology in the OSER assay can still cause aggregation artifacts under some conditions. For example, aggregation of several mCherry fusions can be observed in *Escherichia coli* (*24*–*26*). It is therefore likely that the aggregation properties of FPs vary from one model organism to another and that these properties have to be evaluated in the model organism of interest.

**Fig. 1.**
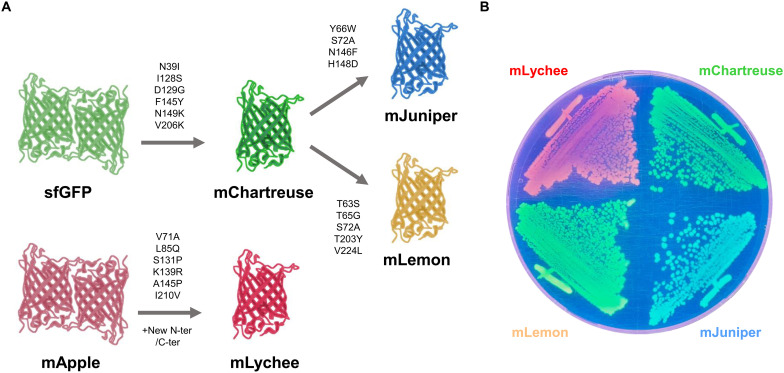
Novel FPs detailed in this study. (**A**) Flow chart of FPs developed in this study. Each arrow shows a development step with its associated substitutions. New N terminus (N-ter)/C terminus (C-ter) refers to the replacement of mApple N and C termini with those of mCherry2C (*27*). (**B**) Images of bacterial colonies carrying pNF02 vectors encoding each indicated FPs, transilluminated with ultraviolet light.

Here, we used site-directed mutagenesis to engineer high-performance FPs. We report the development of four new FPs that offer superior brightness and photostability while being truly monomeric. We developed mChartreuse from sfGFP by introducing six mutations (N39I, I128S, D129G, F145Y, N149K, and V206K) that enhanced brightness, photostability, and monomericity (Fig. 1). We further developed mChartreuse into mJuniper, a cyan variant, with the addition of four mutations (Y66W, S72A, N146F, and H148D), and into mLemon, a yellow variant, with the addition of five mutations (T63S, T65G, S72A, T203Y, and V224L) (Fig. 1, A and B). Compared to state-of-the-art cyan FP (CFP) and yellow FP (YFP), these two FPs offer superior dispersibility and photostability with similar brightness (Table 1). While engineering RFPs, we identified S131P, a substitution that eliminates oligomerization of supposedly monomeric DsRed derivatives in *E. coli*. By combining S131P with brightness-enhancing mutations (V71A, L85Q, K139R, A145P, and I210V), we evolved mApple into mLychee, a bright, monomeric, and photostable RFP (Fig. 1, A and B, and Table 1). We therefore offer a palette of novel high-performance FPs tested in *E. coli,* with superior properties for in vivo time lapse imaging.

**Table 1.** Properties of purified FPs. Columns show means (±SD when applicable) with units in parenthesis: Fluorescence protein (FP), absorbance (Abs) peak, emission (Em) peak, extinction coefficient (EC), QY, brightness (product of EC and QY), p*K*_a_, and maturation half-time.

FP	Abs peak (nm)	Em peak (nm)	EC (mM^−1^ cm^−1^)	QY	Brightness	p*K*_a_	Maturation (min)
mChartreuse	487	510	71 ± 3	0.75 ± 0.02	53 ± 3	4.9 ± 0.3	4.9 ± 0.6
mJuniper	434	475	32 ± 1	0.43 ± 0.04	14 ± 2	4.7 ± 0.1	1.7 ± 1.1
mLemon	514	526	125 ± 5	0.74 ± 0.03	93 ± 6	4.8 ± 0.1	12.0 ± 1.1
mLychee	568	596	80 ± 4	0.50 ± 0.02	40 ± 3	6.3 ± 0.1	36.4 ± 2.2

## RESULTS

### Site-directed mutagenesis improves all properties of sfGFP

**Fig. 2.**
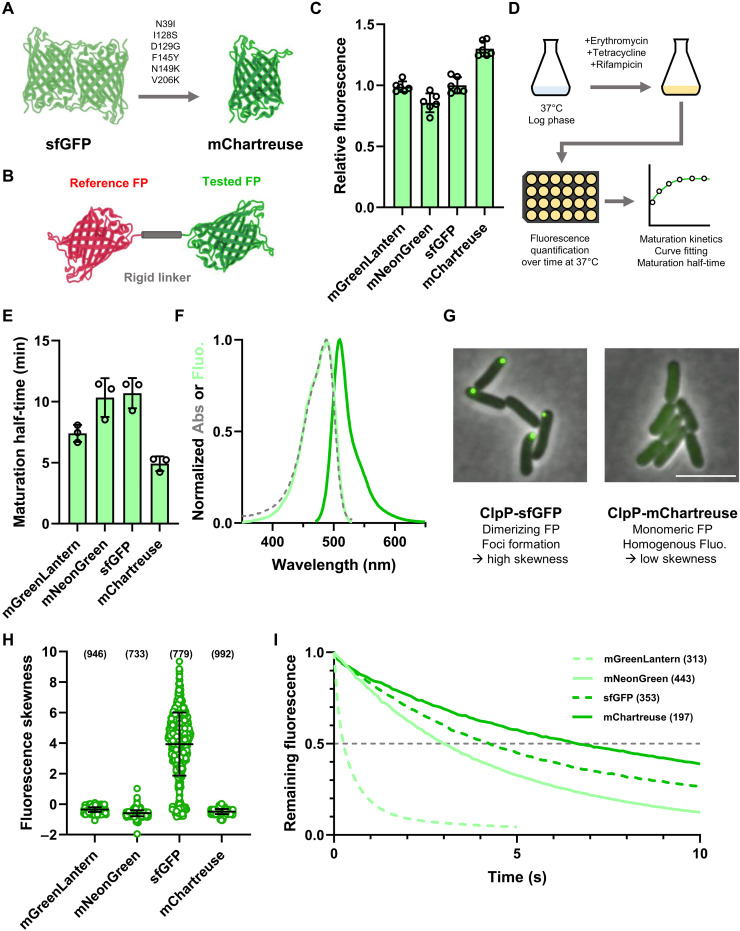
Characterization of the mChartreuse GFP. (**A**) Mutations introduced in sfGFP yielding mChartreuse. (**B**) Brightness quantification system. Ratiometric measurement of the tested FP normalized to that of a fused reference FP enables to offset variations in FP signal due to differences in expression levels. (**C**) In vivo brightness quantification of GFPs. Fluorescence was measured in exponentially growing cultures and normalized to that of mCherry. Data show mean and SD of six independent replicates. (**D**) Description of the maturation assay. Exponentially growing cells were treated with protein synthesis inhibitors, enabling the maturation of dark fluorophores. Maturation kinetics were measured from this point and fitted to a pseudo–first-order kinetic, from which maturation half-times were computed. (**E**) Maturation half-times of GFPs. Data show mean and SD of three independent replicates. (**F**) Spectral properties of mChartreuse. Dashed line shows absorbance spectrum, light-colored line shows excitation spectrum, and dark-colored line shows emission spectrum. Data are shown as the mean of three independent acquisitions. Abs, absorbance; Fluo, fluorescence. (**G**) Representative images of ClpP fusions. Fusion of ClpP with a dimeric FP (sfGFP) causes substantial aggregation, leading to foci formation and high fluorescence skewness. Fusion with a monomeric FP (mChartreuse) does not cause aggregation, leading to homogenous fluorescence distribution and low fluorescence skewness. Scale bar 5 µm. (**H**) Fluorescence skewness of single cells expressing ClpP-FP fusions. Bars show the mean and SD. Data were acquired from three independent experiments, with the total numbers of analyzed bacteria shown in parentheses. (**I**) Photostability of FP signals in live bacteria illuminated with 100-ms steps and normalized to intensity at time 0. Data were acquired from three independent experiments, with the total numbers of analyzed bacteria shown in parentheses.

We first aimed to obtain an improved GFP derived from the well-characterized avGFP scaffold. We chose sfGFP as a starting template due to its improved folding, fast maturation, high brightness, and broad use in bacterial models (*5*, *22*). We first introduced the V206K monomerizing mutation and the F145Y photostability-improving mutation, as previously described for the construction of monomeric superfolder GFP 2 (msGFP2) (*27*). To further improve the brightness of this starting template, we used high-throughput flow cytometry to screen several mutations found in bright avGFP derivatives. After four rounds of site-directed mutagenesis, we identified our brightest variant containing mutations N39I, I128S, and D129G from the Achilles YFP (*28*) and N149K from the Emerald GFP (*29*) (Fig. 2A and fig. S1). We called this variant mChartreuse based on the similarly colored liquor from southeast France.

Purified mChartreuse displayed an extinction coefficient of 71 mM^−1^ cm^−1^ and a QY of 0.75 (Table 1). However, evaluating in vivo brightness is a better indicator of FP performance in living systems. By fusing mChartreuse and other GFPs with mCherry, we can evaluate the in vivo brightness of these FPs in growing cells by normalizing the green fluorescent signal to that of mCherry, therefore allowing us to offset any variation in the expression level of these constructs (Fig. 2B) (*19*). Our results show that mChartreuse is 30% brighter than sfGFP in *E. coli* (Fig. 2C). mChartreuse is also brighter than mNeonGreen and mGreenLantern, two bright and top-performing GFPs that will be used hereafter as comparison points to sfGFP and mChartreuse (Fig. 2C) (*15*, *30*).

To assess how well mChartreuse matured in exponentially growing *E. coli*, we quantified the maturation half-time of mChartreuse and other GFPs by shutting down protein synthesis using a cocktail of protein synthesis inhibitors (erythromycin, tetracycline, and rifampicin), followed by measuring fluorescence maturation kinetics after protein synthesis shutoff (Fig. 2D). In this setup, protein synthesis inhibition arrests fluorophore biosynthesis, allowing remaining dark unmatured fluorophores to fully mature over time (fig. S2A). By fitting a pseudo–first-order kinetics curve to the maturation data, we determined the maturation half-time of mChartreuse to be 4.9 min, faster than its sfGFP parent (10.7 min) or than mNeonGreen (10.3 min) or mGreenLantern (7.4 min) (Table 1 and Fig. 2E).

The excitation and emission spectra of untagged mChartreuse were determined after three-phase partition (TPP) purification (*31*). mChartreuse displayed a broad absorbance peak with a maximum at 487 nm, while its emission peaked at 510 nm, similar to that of sfGFP or EGFP (Table 1 and Fig. 2F) (*5*, *6*). The fluorescence of mChartreuse displays a p*K*_a_ of 4.9 (where *K*_a_ is the acid dissociation constant), showing that it is resistant to fluctuations in cytosolic pH (Table 1 and fig. S3).

We evaluated the dispersibility and oligomerization of mChartreuse and other GFPs by fusing them to the ClpP protease as previously described (*26*). In this system, oligomeric FPs cause cooperative clustering of ClpP homotetradecamers into fluorescent foci, while monomeric FPs do not perturbate the homogenous cytosolic localization of ClpP (*26*). To offer an unbiased quantification of foci formation by these ClpP fusions, we measured the skewness of pixel intensity distributions in a single bacterium as a proxy for fluorescence inhomogeneity (*32*). As previously described, fusion of ClpP with sfGFP (a dimerizing FP) induced foci formation, exemplified by the high fluorescence skewness observed in these cells (Fig. 2, G and H). On the other hand, ClpP fusions with mChartreuse, mNeonGreen, and mGreenLantern all displayed homogeneous fluorescence as shown by the low fluorescence skewness values of these fusions (Fig. 2, G and H). Our results therefore show that mChartreuse is monomeric in *E. coli*.

Because photostability is a critical parameter for time-lapse imaging, we evaluated the time required to bleach mChartreuse to half its original fluorescence intensity (*t*_1/2_) by time-lapse fluorescence microscopy. Of tested GFPs, mChartreuse displayed the highest photostability (*t*_1/2_ = 6.7 s), followed by sfGFP (*t*_1/2_ = 4.3 s), mNeonGreen (*t*_1/2_ = 3.0 s), and, lastly, mGreenLantern (*t*_1/2_ = 0.3 s) (Fig. 2I). Conclusively, the evolution of mChartreuse improved all aspects of sfGFP (i.e., brightness, maturation, dispersibility, and photostability), making it a superior choice for all applications.

### Mutagenesis of mChartreuse yields photostable monomeric CFPs and YFPs

**Fig. 3.**
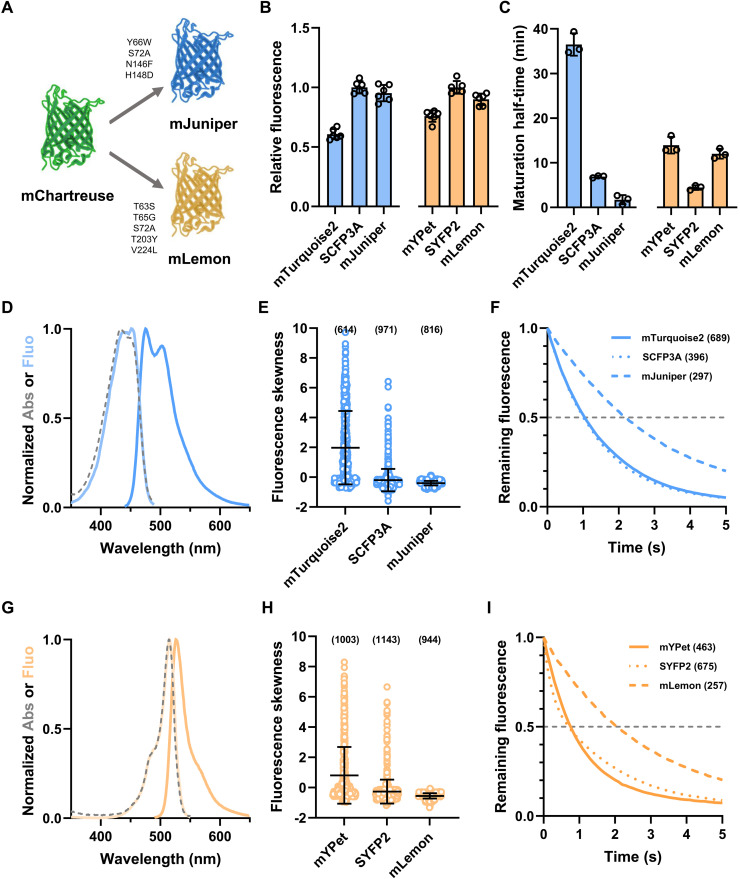
Development and characterization of cyan and yellow variants of mChartreuse. (**A**) Mutations introduced in mChartreuse to obtain the mJuniper cyan variant and the mLemon yellow variant. (**B**) In vivo brightness quantification of CFP and YFP. Fluorescence was measured in exponentially growing cultures and normalized to that of mCherry (for CFPs) or mTurquoise2 (for YFPs). Data show mean and SD of six independent replicates. (**C**) Maturation half-times of CFP and YFP. Data show mean and SD of three independent replicates. (**D** and **G**) Spectral properties of mJuniper (D) and mLemon (G). The dashed line shows the absorbance spectrum, the light-colored line shows the excitation spectrum, and the dark-colored line shows the emission spectrum. Data are shown as the mean of three independent spectrum acquisitions. (**E** and **H**) Fluorescence distribution skewness of single cells expressing ClpP-CFP (E) or ClpP-YFP (H) fusions. Bars show the mean and SD. Data were acquired from three independent experiments, with the total numbers of analyzed bacteria shown in parentheses. (**F** and **I**) Photostability of CFP signals (F) and YFP signals (I) in live bacteria illuminated with 100-ms steps and normalized to intensity at time 0. The dashed gray line shows the cutoff at which *t*_1/2_ was calculated. Data were acquired from three independent experiments, with the total numbers of analyzed bacteria shown in parentheses.

Since mChartreuse is a bright and photostable variant of sfGFP, we assessed whether this FP could be engineered into cyan and yellow variants with superior properties. We developed a cyan variant of mChartreuse by introducing the Y66W cyan-determining mutation (*8*), followed by the S72A, N146F, and H148D mutations found in mTurquoise2 (Fig. 3A and fig. S1) (*33*). This variant, named “mJuniper,” was compared to super CFP 3A (SCFP3A) and mTurquoise2, two top-performing CFPs (*33*, *34*). The brightness of mCherry-fused mJuniper was equivalent to that of SCFP3A, with both these FPs being roughly 50% brighter mTurquoise2 (Fig. 3B). mJuniper displayed unusually fast maturation kinetics, with a maturation half-time of 1.7 min, faster than SCFP3A (6.9 min) or mTurquoise2 (36.5 min) (Table 1, Fig. 3C, and fig. S2B). mJuniper showed an absorbance peak at 434 nm and an emission peak at 475 nm as described for SCFP3A and mTurquoise2 (Table 1 and Fig. 3D) (*33*, *34*). The p*K*_a_ of mJuniper is 4.7, showing that it can withstand changes in cytosolic pH (Table 1 and fig. S3). Fusions of ClpP with mTurquoise2 and SCFP3A both lead to foci formation in a substantial number of cells, while fusion of ClpP with mJuniper yielded homogenous fluorescence, showing that mJuniper is a true monomer (Fig. 3E). Moreover, mJuniper displayed higher photostability (*t*_1/2_ = 2.2 s) compared to mTurquoise2 (*t*_1/2_ = 1.1 s) and SCFP3A (*t*_1/2_ = 1.1 s) (Fig. 3F). Therefore, mJuniper is a bright and truly monomeric CFP with superior maturation and photostability in *E. coli*.

A yellow variant of mChartreuse was engineered by first introducing the T203Y yellow-determining mutation, followed by the T65G and S72A found in most yellow derivatives of avGFP (Fig. 3A and fig. S1) (*9*). We also added the T63S mutation from mGold and the V224L from YFP for energy transfer (YPet) (Fig. 3A) (*35*, *36*). We called this variant “mLemon” and compared it to mYPet (YPet A206K) and Super YFP 2 (SYFP2, mVenus L68V), two top-performing YFPs (*34*, *36*). SYFP2 and mLemon were both brighter than mYPet, with mLemon showing 90% of the brightness of SYFP2 (Fig. 3B). mLemon matured at a similar rate as mYPet (12.0 and 13.9 min, respectively), both maturing slower than SYFP2 (4.4 min) (Table 1, Fig. 3C, and fig. S2C). The spectrum of mLemon was similar to that of other YFPs (*34*, *36*), with narrow absorbance and emission peaks at 514 and 526 nm, respectively (Table 1 and Fig. 3G). mLemon showed high resistance to acid with a p*K*_a_ of 4.7 (Table 1 and fig. S3). While some YFPs can be quenched by chloride ions (*37*), we show mLemon fluorescence to be insensitive to increases in sodium chloride concentration (fig. S4). ClpP-mLemon displayed homogenous fluorescence, while ClpP-mYPet and ClpP-SYFP2 both showed aggregation, showing that mLemon is monomeric (Fig. 3H). mLemon displayed more than twice the photostability (*t*_1/2_ = 2.1 s) of mYPet (*t*_1/2_ = 0.8 s) and SYFP2 (*t*_1/2_ = 0.8 s) (Fig. 3I). Therefore, mLemon offers higher photostability and lower aggregation compared to other YFPs while still retaining high brightness.

### Mutagenesis enables monomerization and enhancement of the mApple RFP

**Fig. 4.**
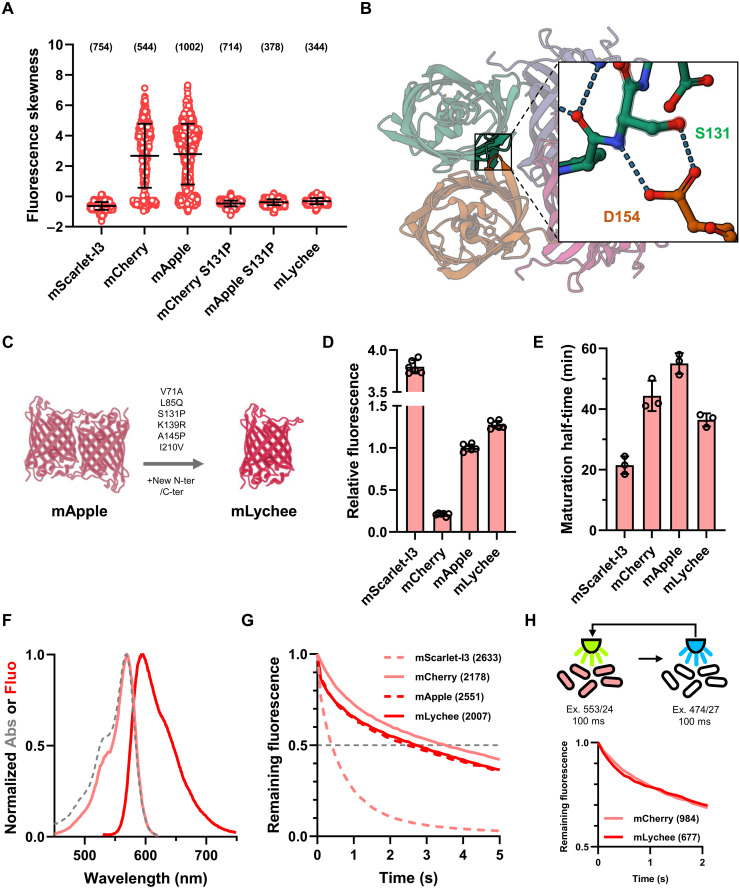
Development and characterization of the mLychee RFP. (**A**) Fluorescence distribution skewness of single cells expressing ClpP-FP fusions. Bars show the mean and SD. Data were acquired from three independent experiments, with the total numbers of analyzed bacteria shown in parentheses. (**B**) Location of S131 in the AB dimerization interface of a DsRed tetramer (Protein Data Bank 1ZGO) shown in ribbon representation. The inlet shows the interaction between S131 and D154 located in trans at the atomic level, with putative hydrogen bonds shown as dashed lines. (**C**) All six mutations introduced in mApple to obtain mLychee. (**D**) In vivo brightness quantification of RFPs. Fluorescence was measured in exponentially growing cultures and normalized to that mTurquoise2. Data show mean and SD of six independent replicates. (**E**) Maturation half-times of RFPs. Data show mean and SD of three independent replicates. (**F**) Spectral properties mLychee. The dashed line shows the absorbance spectrum, the light-colored line shows the excitation spectrum, and the dark-colored line shows the emission spectrum. Data are shown as the mean of three independent spectrum acquisitions. (**G**) Photostability of FP signals in live bacteria illuminated with 100-ms steps and normalized to intensity at time 0. The dashed gray line shows the cutoff at which *t*_1/2_ was calculated. Data were acquired from three independent experiments, with the total numbers of analyzed bacteria shown in brackets. (**H**) Short-term photostability of mCherry and mLychee signals under alternating green (555 nm) and blue (474 nm) illumination. Data were acquired from three independent experiments, with the total numbers of analyzed bacteria shown in parentheses. Ex., excitation.

We also sought to engineer a bright and photostable mRFP. While mScarlet-I3 was monomeric and offered unrivaled brightness, it showed lower photostability compared to other RFPs (*20*). On the other hand, mFruits such as mCherry and mApple offered higher photostability (*13*, *14*). However, previous data show that mFruits aggregate when fused to ClpP in *E. coli* (*26*). Our data show that fusions of mCherry and mApple with ClpP had a high propensity to form foci, confirming that these FPs are not monomeric (Fig. 4A). On the other hand, ClpP–mScarlet-I3 did not form foci, indicating that this FP is truly monomeric (Fig. 4A). Since mScarlet-I3 was derived from an mFruit-based synthetic template, we introduced mutations specific to mScarlet-I3 into mApple and then screened for mutations conferring a nonaggregative behavior in ClpP fusions. The S131P mutation (numbering relative to DsRed) abolished foci formation in ClpP fusions with mApple or mCherry (Fig. 4A). In DsRed tetramers, S131 is part of the AB tetramer interface between two DsRed protomers, with the hydroxyl sidechain and the amide nitrogen of S131 each implicated in a hydrogen bond with the carboxyl sidechain of D154 located in trans (Fig. 4B). It is therefore likely that the substitution of S131 with a proline prevents H-bond formation with D154 and eliminates residual dimerization of mFruits in *E. coli*. We used mApple-S131P as a starting template to engineer a brighter RFP. During the previous engineering of mFruits, the original N and C termini of DsRed were replaced with those of avGFP (*14*). While this allowed proper localization of some fusions, restoration of DsRed-like termini was shown to improve brightness and relieve RFP-induced cytotoxicity (*20*, *27*). We therefore synthetized a codon-optimized mApple template, replaced the MVSKGEENNM N terminus and TGGMDE C terminus of mApple with the MDSTE N terminus and GSQGGSGGS C terminus of mCherry2C (Fig. 4C and fig. S5) (*27*). We subsequently performed rounds of site-directed mutagenesis to identify mutations conferring increased in vivo brightness. Mutations V71A, L85Q, K139R, A145P, and I210V were incorporated in our final variant, named mLychee (Fig. 4C and fig. S5).

The mLychee RFP is 28% brighter than mApple and six times brighter than mCherry under excitation at 565 nm, while mScarlet-I3 outperforms other FPs, being more than thrice as bright as mApple (Fig. 4D). mLychee showed a maturation half-time of 36.4 min, faster than its mApple parent (55.0 min) or than mCherry (44.4 min) but slower than mScarlet-I3 (21.5 min) (Table 1, Fig. 4E, and fig. S2D). Similarly to what was published for mScarlets and mApple (*13*, *19*, *20*), mLychee displays an absorbance peak at 568 nm, an emission peak at 596 nm, and a p*K*_a_ of 6.3 (Table 1, Fig. 4F, and fig. S3). mCherry displayed the highest photostability (*t*_1/2_ = 3.6 s), while mScarlet-I3 bleached the quickest (*t*_1/2_ = 0.4 s) (Fig. 4G). mApple and mLychee displayed complex bleaching behaviors, with an abrupt loss of 15% of their signal after the first 100-ms exposure, followed by slow bleaching kinetics similar to that of mCherry (*t*_1/2_ = 2.7 and 2.8 s, respectively) (Fig. 4G). Previous results showed that this fast initial bleaching of mApple was a photochromic phenomenon reversible either by time or by illuminating this fluorophore with blue light (*13*). We therefore quantified the bleaching of mLychee by alternating illuminations between 555 and 474 nm (Fig. 4H). Under these conditions, the fast initial bleaching of mLychee was practically eliminated, yielding initial bleaching kinetics similar to that of mCherry (Fig. 4H).

### Novel FP derivatives enhance applications in *E. coli*

**Fig. 5.**
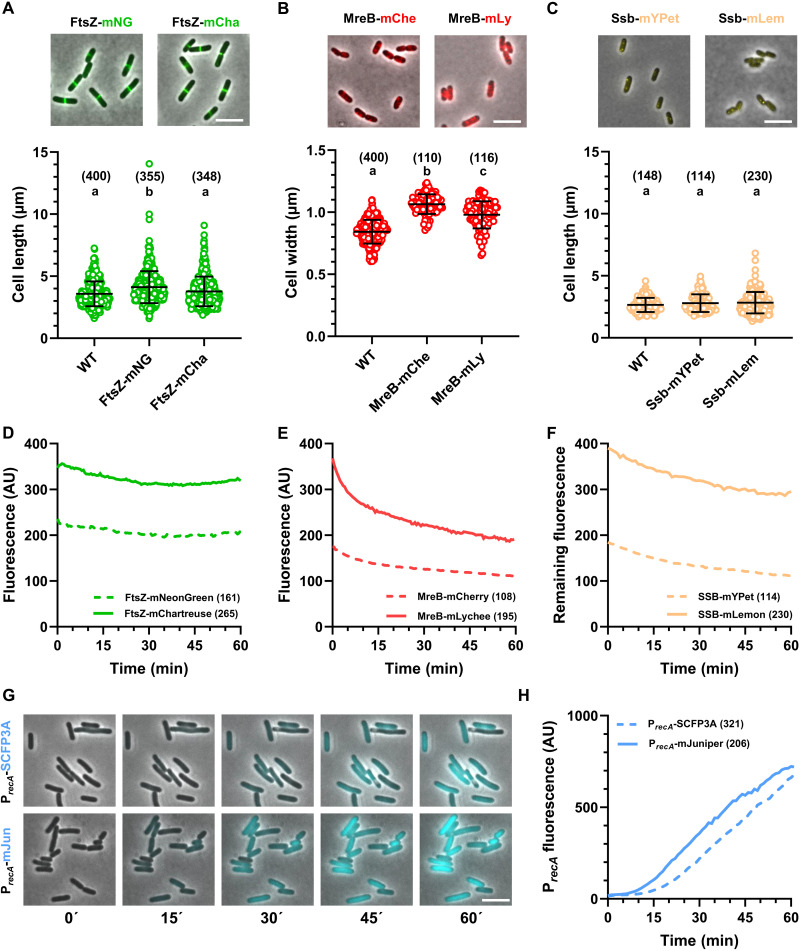
Applications of novel FP derivatives in *E. coli*. (**A** to **C**) Top: Representative images of cells encoding FtsZ-mNeonGreen (mNG) and FtsZ–mChartreuse (mCha) (A), MreB–mCherry (mChe) and MreB–mLychee (mLy) (B) grown to an optical density (OD) of 0.2 to 0.3 in M9GC medium, and Ssb-mYPet and Ssb–mLemon (mLem) (C) grown to an OD of 0.2 to 0.3 in M9 glucose medium. Scale bars, 5 μm. Bottom: Distribution of cell length in wild-type (WT) TB28 cells or cells encoding FtsZ-mNeonGreen or FtsZ-mChartreuse (A), cell width in wild-type, MreB-mCherry, or MreB-mLychee (B), cell length in wild-type, Ssb-mYPet, or Ssb-mLemon (C), with the number of analyzed cells in parentheses. Abc, statistical groups (*P* < 0.001, Dunn’s multiple comparison test). (**D** to **F**) Photostability of translational fusions, showing raw intensities of FtsZ-mNeonGreen and FtsZ-mChartreuse (D), MreB-mCherry and MreB-mLychee (E), and Ssb-mYPet and Ssb-mLemon (F) illuminated every 30 s [(D) and (E)] or 60 s (F), with the total number of analyzed bacteria shown in parentheses. AU, arbitrary units. (**G**) Representative images of cells carrying a *recA* transcriptional reporter fused with either SCFP3A or mJuniper. Cells were grown in M9GC to an OD of 0.2 to 0.3, spotted on M9GC agarose pads containing ciprofloxacin (15 μg/ml) and imaged every minute. Scale bar, 5 μm. (**H**) Time-course analysis of *recA* transcriptional reporters showing the evolution of cyan fluorescence of cells as in (D), with the number of analyzed cells in parentheses.

To assess the performance of our novel FPs in a biologically relevant context, we first constructed fusions with mChartreuse or mLychee and compared to corresponding standards mNeonGreen and mCherry, respectively. We fused mChartreuse and mNeonGreen to filamentation temperature sensitive Z (FtsZ), an essential bacterial homolog of tubulin that assembles into a ring at the future division septum, while mLychee was fused to murein formation cluster E (MreB), a bacterial homolog of actin that controls cell shape. These essential cytoskeletal proteins are notoriously difficult to fuse to FPs, which must be inserted in internal loops of FtsZ and MreB in so-called “sandwich” fusions to produce viable strains containing functional reporter fusions as a sole *ftsZ* or *mreB* copies (*38*, *39*). Nevertheless, these fusions are known to cause morphological abnormalities, with FtsZ fusions yielding abnormally long cells (*39*, *40*) and MreB fusions leading abnormally wide cells (*38*, *41*). Cells encoding FtsZ-mNeonGreen were notably longer (4.12 ± 1.29 μm) than wild-type cells (3.58 ± 0.99 μm) (Fig. 5A). On the other hand, cells encoding FtsZ-mChartreuse displayed a length distribution (3.77 ± 1.19 μm) close to that of wild-type cells, suggesting that fusion with mChartreuse has a lower impact on FtsZ functionality than fusion with mNeonGreen (Fig. 5A). Similarly, fusion of MreB with mCherry causes a notable increase in cell width (1.07 ± 0.08 μm) compared to wild-type cells (0.84 ± 0.10 μm) (Fig. 5B). Fusion of MreB with mLychee leads to an intermediate increase in cell width (0.98 ± 0.11 μm), suggesting that fusion with mLychee had a lesser impact of MreB functionality than fusion with mCherry (Fig. 5B). Time-lapse analysis with 30-s intervals and equal exposure times reveals that the mean cellular fluorescence intensities of cells expressing FtsZ-mChartreuse and MreB-Lychee consistently remain higher than those of cells expressing FtsZ-mNeonGreen and MreB-mCherry, respectively, throughout a 60-min duration (Fig. 5, D and E). Similarly, mLemon was fused to the essential single-stranded binding protein (Ssb) and imaged in minimal medium. Ssb enables the localization of replication forks, and its impairment results in cell filamentation. We observed that Ssb-mLemon displayed normal cell shape and higher intensity than an Ssb-mYPet fusion over the course of a 60-min time lapse (Fig. 5, C and F). We also assessed whether the superior maturation rate of mJuniper enables earlier detection of a transcriptional reporter compared to a reporter constructed with the slower-maturing SCFP3A. To do so, we have constructed a reporter of the SOS response by fusing the *recA* promoter (P*_recA_*) to SCFP3A or mJuniper. We subsequently followed induction of this reporter when cells were grown on agarose pads containing ciprofloxacin (15 μg/ml) for 2 hours (Fig. 5, G and H). While induction of P*_recA_* was detectable with SCFP3A or mJuniper, the P*_recA_*-mJuniper reporter enabled detection of a transcriptional response earlier (~10 min) than with P*_recA_*-SCFP3A (Fig. 5, G and H), showing that the fast maturation rate of mJuniper makes it a superior reporter to follow transcription kinetics. These results demonstrate that our FPs offer enhanced detection over extended time-lapse durations, while enabling imaging with reduced exposure times compared to standard FPs, thereby preserving samples from phototoxicity.

## DISCUSSION

Here, we introduce a novel palette of FPs tailored for bacterial applications. This toolkit that includes mChartreuse, mJuniper, mLemon, and mLychee represents a notable advancement in time-lapse imaging with its improved properties compared to traditional and commonly used FPs. The enhanced photostability and minimal aggregation of these novel proteins make them highly suitable fusion tags, and their spectral characteristics enable straightforward integration into existing research workflows without requiring specialized equipment. Contrasting with other modern bright FPs such as mNeonGreen or StayGold, mChartreuse and its derivatives were engineered from sfGFP, a well-folded GFP that is broadly described and used in bacterial imaging studies. Applications that currently use sfGFP can therefore be quickly adapted to use mChartreuse, mJuniper, and mLemon. Further testing of these novel FPs as fusion tags in *E. coli* and other bacterial species will enable us to assess how well these fusions preserve native localization and activities of essential prokaryotic proteins. The high brightness and photostability of our FP derivatives also enable bacterial time-lapse imaging with lower doses of illuminations, thereby reducing phototoxicity.

Our findings confirm that the F145Y mutation improves the photostability of sfGFP derivatives, consistent with previous research (*27*). However, the relationship between oligomerization and residue substitution appears more intricate. To examine FP oligomerization in *E. coli*, we developed a quantitative method based on the skewness in fluorescence distribution of ClpP fusions within a single bacterium, enabling unbiased and robust quantification of FP aggregation and foci formation. Our results confirm that the V206K mutation abolishes oligomerization in sfGFP derivatives (*27*). However, since the A206K mutation is also present in mVenus and mYPet, which tend to aggregate in the ClpP assay (*26*), this suggests that aggregation behavior is influenced by factors beyond this mutation, such as superfolder mutations present in our initial sfGFP template (S30R, F99S, N105T, and I171V) or mutations that were introduced during the development of mChartreuse (N39I, I128S, D129G, F145Y, and N149K).

Previous research showed that mCherry and mApple are monomers in mammalian cells when assayed using the OSER approach (*19*, *21*). However, fusions of mCherry with ClpP, IbpA (inclusion body protein A), or RpoS (RNA polymerase sigma factor S) in *E. coli* all caused substantial aggregation (*24*–*26*), suggesting that RFPs derived from *Discosoma* sp. are not monomeric in bacterial cells. The reasons for these discrepancies are not known but might stem from differences in cytosolic composition and crowding between mammalian and bacterial cells or in differences of sensitivity between the OSER and ClpP assays. Nevertheless, substitution of serine 131 in mApple by a proline abolishes the aggregating behavior of the mLychee variant developed from mApple. While mLychee is dimmer than mScarlet-I3, it compensates for this lack of brightness with robust photostability. mLychee would therefore be more appropriate for time-lapse applications, while mScarlet-I3 would remain preferable for endpoint methods (e.g., epifluorescence microscopy snapshots and flow cytometry) where photobleaching due to repeated excitation is not an issue.

We also report straightforward and quantitative assays to evaluate FP oligomerization and maturation rate. On the basis of the work of Landgraf *et al.* (*26*), we propose the use of ClpP fusions as a standardized assay to measure FP oligomerization in *E. coli*. Our approach refines Landgraf’s method using a more user-friendly complementation approach, which consists of introducing an FP-tagged and plasmid-encoded copy of *clpP* in a *clpP* background, instead of in situ tagging of endogenous *clpP* using lambda red recombineering. FP oligomerization and foci formation are quantified using fluorescence skewness as a proxy, which can be automatically performed on batches of images using the MicrobeJ suite. To quantify FP maturation rate, we adapted the protein synthesis shutdown assay developed by Balleza *et al.* (*22*) for use in automated microplate reader, instead of microscopy coupled to microfluidics, which therefore renders this assay more accessible and less work-intensive. These two assays do not rely on uncommon equipment or techniques and can therefore easily be implemented in new laboratories and used to assess FP oligomerization and maturation of interesting novel FP candidates.

Although these novel FPs were initially developed for bacterial applications, they have the potential to substantially enhance imaging techniques in eukaryotic cells as well. One major consideration in transferring these FPs to eukaryotic systems is their compatibility with the cellular environment, including cytosolic composition, protein expression, and folding dynamics, which differ notably from bacterial cells. Therefore, optimizing these FPs to eukaryotic systems would require adaptations such as codon usage optimization for efficient expression, as well as potential adjustments to accommodate eukaryotic posttranslational modifications and cellular environments.

## MATERIALS AND METHODS

### Strains, cultures, and microbiological procedures

*E. coli* TB28 (MG1655 Δ*lacIZYA*) (*42*) was used in all experiments except those involving ClpP fusions, which were performed in strain LY3581 (MG1655 Δ*clpP::FRT*). Strain LY3581 was constructed by P1 transduction of the *clpP* deletion from strain JW0427 (*43*). All experiments were performed by growing cells in M9 medium (referred to as M9GC): Na_2_HPO_4_.7H_2_O (7 g/liter), KH_2_PO_4_ (3 g/liter), NaCl (0.5 g/liter), NH_4_Cl (1 g/liter), 1 mM MgSO_4_, and 80 μM CaCl_2_ supplemented with 0.2% glucose, 0.4% casamino acids, and thiamine hydrochloride (0.4 mg/liter). Overnight cultures were inoculated in M9GC containing appropriate antibiotics [chloramphenicol (10 μg/ml) for pFN01 or kanamycin (25 μg/ml) for pCLP derivatives] from colonies, followed by 1000× dilution in fresh M9GC medium without antibiotics. Cells were then grown to exponential phase, to a turbidity [optical density at 600 nm (OD_600_)] of 0.2 to 0.3 at which experiments were performed. Fluorescence in bulk cultures was measured in 96-well plates.

### Fluorescence measurements in bulk cultures

All fluorescence measurements were performed on a Spark plate reader (Tecan) using a 5-nm bandpass monochromator. Cyan fluorescence was excited at 450 nm and collected at 480 nm, green fluorescence was excited at 480 nm and collected at 510 nm, yellow fluorescence was excited at 510 nm and collected at 540 nm, and red fluorescence was excited at 565 nm and collected at 595 nm.

### Molecular cloning procedures and strain construction

All plasmid constructs were obtained by Gibson assembly unless specified otherwise and are detailed in the Supplementary Materials (table S1). All primers used to construct these vectors are detailed in the Supplementary Materials (table S2). All enzymes were purchased from New England Biolabs. Polymerase chain reactions were performed using PrimeSTAR Max (Takara). Synthetic genes for mApple with modified N and C termini and mGreenLantern were ordered from Integrated DNA Technologies.

pNF02 vectors used for unfused FP expression were constructed by amplifying a mini-F backbone from pNF02–mScarlet-I (*24*) using primers bbpNF02 F and R. sfGFP was introduced in pNF02 using primers sfGFP02 F and R, while mApple with new N and C termini (mAppleNC) was directly inserted as a synthetic gene. Mutations to construct mChartreuse were introduced in pNF02-sfGFP using primers N39I F and 39 R, D129G F and I128S, N149K F and Y145F R, and V206K F and 206 R. mJuniper was generated from pNF02-mChartreuse using primers S72A F and Y66W and H148D F and N146F R, while mLemon was constructed using primers S72A F and S65GT63S F, V206K F and T203Y, and V224L F and 221 R. mLychee was obtained from pNF02-mAppleNC using primers V71A F and L85Q R, S131P F and R, A145P F and K139R R, and 219 F and I210V R.

pFN01 vectors, which encode all tested FPs fused to a reference FP and separated with a rigid linker, were constructed by amplifying a mTurquoise2–linker–mScarlet-I3 fragment from pDress-mTurquoise2-link-mScarlet-I3 (*20*) with primers IpFN01 F and R and a mini-F backbone from pNF02 using VpFN01 F and R. RFP and YFP were amplified using primer pairs 01miscF and R (mCherry, mApple, and SYFP2), 01YPet F and R (mYPet), and 01sfGFP F and R (mLemon) on their respective templates (table S1) (*13*, *20*). To clone green and CFPs, mTurquoise2 was first replaced by mCherry on pFN01 by amplification of the backbone using VmTq2mCh F and R and by amplification of mCherry from pROD62 using ImTq2mCh F and R. Subsequently, FPs were cloned in this vector using primers 01sfGFP F and R (sfGFP, mChartreuse, and mJuniper), 01mNGb F and R (mNG), mGL F and 01sfGFP R (mGreenLantern), and 01misc F and R (mTurquoise2) amplified from their respective templates (table S1) (*20*, *44*). A pFN01-mCherry-SCFP3A was generated by site-directed mutagenesis from pFN01-mCh-mTq2 using VSCFP3A F and R and ISCFP3A F and R.

The pCLP vector used to fuse FPs to ClpP was constructed by amplifying *clpP* and its native promoter using primers clpP F and R. This fragment was digested with Nhe I and Bam HI and cloned in pUA66 (*45*) digested with Avr II and Bam HI. To construct ClpP-FP fusions, FPs were amplified from corresponding pFN01 vectors with primers insCLP F and R and cloned in a pCLP backbone amplified with pCLP F and R. Mutation S131P was introduced on pCLP-mCherry and pCLP-mApple using primers mFruitS131P F and R. pET151 vectors used for untagged FP production were constructed by amplification of a pET151 backbone using primers pET151 F and R and of mChartreuse, mLemon, mJuniper, or mLychee from pNF02 vectors using primers 151FP F and R.

FtsZ and MreB sandwich fusions were constructed by homologous recombination using the pKNG101 suicide plasmid (*46*). Homology arms and FPs were amplified using ad hoc primers (table S2) and assembled in Bam HI–digested pKNG101 to obtain pKNG101-ftsZ-mCha and pKNG101-mreB-mLychee. mNeonGreen was replaced with mChartreuse using primers bbftsZ F/R and ftsZmNG F/R to obtain pKNG101-ftsZ-mNG.

Transcriptional fusions of P*_recA_* were constructed by replacing GFPmut2 from pUA139-recA (*45*) with mJuniper using primers pUA66 fluoswap F/R and mJunUA66 F/R, yielding pUArecA-mJun. To obtain pUArecA-SCFP3A, mJuniper was replaced with SCFP3A using primers pUA66 fluoswap F, SCFP3Aopt R/F, and CFPUA66 R, yielding pUArecA-SCFP3A.

### Maturation kinetics measurements

TB28 cells carrying pFN01 vectors were grown to an OD_600_ of 0.2 to 0.3. A preheated 24-well plate was prepared with each well containing 500 μl of M9GC medium supplemented with erythromycin (200 μg/ml), tetracycline hydrochloride (20 μg/ml), and rifampicin (20 μg/ml). Culture (500 μl) was mixed in each well, after which fluorescence acquisition was started with steps of 30 min for slow-maturing FPs (RFPs and mTurquoise2) or 5 min for fast-maturing FPs, with heating (37°C) and shaking every 5 min. A pseudo–first-order kinetic curve [*F*_(*t*)_ = (*F*_max_ − *F*_0_) × (1 − *e*^−k*t^ + *F*_0_)] was fitted to each replicate using the least squares method, with *F*_max_ (fluorescence at plateau) and *k* (kinetic constant) as parameters, *t* (time) and *F*_(*t*)_ (fluorescence) as the variables, and *F*_0_ (fluorescence at time 0) being experimentally determined. Maturation half-time was calculated from these fitted kinetics as the time required to mature half of the remaining fluorescence signal [*F*_(*t*)_ = (*F*_max_ + *F*_0_) / 2].

### Biochemical and biophysical procedures

Untagged FPs were purified from BL21(DE3) cells carrying pET151 FP expression vectors. Fifty milliliters of cultures grown in LB [yeast extract (5 g/liter), tryptone (10 g/liter), and NaCl (5 g/liter)] at 30°C were brought to an OD_600_ of 0.6, after which FP production was induced overnight by the addition of 1 mM isopropyl-β-d-thiogalactopyranoside. Cells were subsequently pelleted and resuspended in 500 μl of lysis buffer [50 mM tris-HCl (pH 8.0), 300 mM NaCl, and 5% glycerol] and beaten with ~300 μl of 100-μm glass beads (5 min, 30 Hz) using a TissueLyser II (QIAGEN). Lysates were separated from beads by piercing the bottom of the tube and by collecting the liquid phase in a bigger tube by centrifugation. After clearing by centrifugation, lysates are processed by TPP purification (*31*). A first TPP step was performed with 20% ammonium sulfate saturation and 1 vol of *t*-butanol at room temperature. After phase separation by centrifugation, the aqueous phase of this TPP was subjected to a second step with 60% ammonium sulfate saturation and 2 vol of *t*-butanol at room temperature, from which the interphase was isolated by centrifugation. This interphase was resuspended in 10 mM tris-HCl (pH 8.0) and rid of insoluble impurities by centrifugation. A final desalting step was performed using Sephadex G-25 spin columns (Cytiva). Purified FPs were stored at 4°C in the dark and were left to mature at least 24 hours before experiments. Centrifugation of FP mother liquors was performed before all experiments to remove insoluble interfering impurities. All absorbance measurements were performed using FPs diluted in 10 mM tris-HCl (pH 8.0) to achieve a peak absorbance of 0.2, while all samples for fluorescence measurements were diluted to a peak absorbance of 0.04.

Extinction coefficients were determined as described (*21*), by normalizing the absorbance of FP samples to that of FPs denatured with 1 M NaOH, under the assumption that the molar extinction coefficient of the denatured chromophore at 447 nm (mChartreuse and mLemon) or 457 nm (mLychee) is 44 mM^−1^ cm^−1^. Since mJuniper could not be denatured by strong bases, its concentration was determined by bicinchoninic acid (BCA) assay (Pierce BCA Protein Assay Kit, Thermo Fisher Scientific). QYs were determined as described (*21*), using fluorescein in 0.1 M NaOH (QY = 0.85) (*47*) as a reference for mChartreuse, mLemon, and mJuniper, while rhodamine B in absolute ethanol (QY = 0.49) (*48*) was used as a reference for mLychee.

Buffer titration to determine p*K*_a_ values was performed by mixing FP samples with 1 vol of citrate phosphate borate buffer at a given pH as described (*20*). These buffers were prepared from a solution of 100 mM citric acid, 100 mM boric acid, and 100 mM monosodium phosphate titrated with NaOH. Buffers were left to stabilize 24 hours at room temperature, yielding pH values of 3.01, 3.92, 4.92, 5.93, 7.07, 8.03, 8.90, and 9.94. Each replicate of fluorescence measurements was fitted to a Hill function [*F*_(pH)_ = *F*_max_ / (1 + 10^n*(pKa-pH)^] using the least-squares method, with *F*_(pH)_ (fluorescence at a given pH) and pH as variables and *F*_max_ (fluorescence at plateau), *n* (Hill coefficient), and p*K*_a_ as parameters.

### Microscopy procedures and experiments

Bacteria grown as described in general procedures were sealed on M9GC pads containing 1% agarose using GeneFrames (Thermo Fisher Scientific). Cells were imaged using an Eclipse Ti2 microscope (Nikon) equipped with a Plan Apo λ 100×/1.45 objective, a motorized stage, Z-drift correction (Perfect Focus System, Nikon), heating (Okolab), a solid-state light source (Spectra X, Lumencor), and a scientific complementary metal-oxide semiconductor camera (Orca-Fusion BT, Hamamatsu). Cyan fluorescence was excited using a 438/24 excitation filter and collected using a 482/25 emission filter, green fluorescence was excited using a 474/27 excitation filter and a 515/30 emission filter, and yellow fluorescence was excited using a 509/22 excitation filter and a 544/25 emission filter. RFPs were excited using a 578/21 excitation filter and a 641/75 emission filter for ClpP fusions or with a 553/24 excitation filter for photostability experiments. All FPs were imaged using ad hoc light-emitting diode (LEDs) at 50% power intensity with 100-ms exposure times, except RFP photostability experiments that used 12% power of the 555-nm LED. Images were subsequently processed using MicrobeJ by automatic detection of cells and quantification of single-cell fluorescence parameters (i.e., mean for photostability experiments and skewness for ClpP fusions) (*32*). Photobleaching experiments were performed by bleaching live cells with 100-ms steps using appropriate excitation settings as described above. Photobleaching half-times were determined as the time at which the mean fluorescence of the population dropped below 0.5. Time-lapse experiments on fusions were performed using the following power/exposition parameters: 5%/150 ms (GFP), 5%/200 ms (RFP), 10%/100 ms (YFP), and 1%/200 ms (CFP).
