# Supplementary material for: A palette of bright and photostable monomeric fluorescent proteins for bacterial time-lapse imaging
Source: Sci Adv. 2025 Apr 16;11(16):eads6201. doi: 10.1126/sciadv.ads6201 (PMC12002091; doi:10.1126/sciadv.ads6201)
Supplement: Supplementary file 1 — Figs. S1 to S5 Tables S1 and S2 [file sciadv.ads6201_sm.pdf]

Science Advances
AAAS

Supplementary Materials for

A palette of bright and photostable monomeric fluorescent proteins for bacterial time-lapse imaging

Nathan Fraikin *et al.*

Corresponding author: Christian Lesterlin, christian.lesterlin@inserm.fr; Nathan Fraikin, nathan.fraikin@ibcp.fr

Sci. Adv. **11**, eads6201 (2025)
DOI: 10.1126/sciadv.ads6201

This PDF file includes:

Figs. S1 to S5
Tables S1 and S2

	10	20	30	40	
avGFP	M-SKGEELFT	GVVPILVELD	GDVNGHKFSV	SGEGEGDATY	GKLTLKFICT
mGreenlantern	MVSKGEELFT	GVVPILVELD	GDVNGHKFSV	RGEGEGDATN	GKLTLKFICT
sfGFP	M-SKGEELFT	GVVPILVELD	GDVNGHKFSV	RGEGEGDATN	GKLTLKFICT
mChatreuse	M-SKGEELFT	GVVPILVELD	GDVNGHKFSV	RGEGEGDAT**I**	GKLTLKFICT
mTurquoise2	MVSKGEELFT	GVVPILVELD	GDVNGHKFSV	SGEGEGDATY	GKLTLKFICT
SCFP3A	MVSKGEELFT	GVVPILVELD	GDVNGHKFSV	SGEGEGDATY	GKLTLKFICT
mJuniper	M-SKGEELFT	GVVPILVELD	GDVNGHKFSV	RGEGEGDAT**I**	GKLTLKFICT
mYPet	M-SKGEELFT	GVVPILVELD	GDVNGHKFSV	SGEGEGDATY	GKLTLKLLCT
SYFP2	MVSKGEELFT	GVVPILVELD	GDVNGHKFSV	SGEGEGDATY	GKLTLKLICT
mLemon	M-SKGEELFT	GVVPILVELD	GDVNGHKFSV	RGEGEGDAT**I**	GKLTLKFICT

	50	60 ***	70	80	90
avGFP	TGKLPVPWPT	LVTTFSYGVQ	CFSRYPDHMK	QHDFFKSAMP	EGYVQERTIF
mGreenlantern	TGKLPVPWPT	LVTTLGYGVA	CFARYPDHMK	QHDFFKSAMP	EGYVQERTIS
sfGFP	TGKLPVPWPT	LVTTLTYGVQ	CFSRYPDHMK	QHDFFKSAMP	EGYVQERTIS
mChatreuse	TGKLPVPWPT	LVTTLTYGVQ	CFSRYPDHMK	QHDFFKSAMP	EGYVQERTIS
mTurquoise2	TGKLPVPWPT	LVTTLSWGVQ	CFARYPDHMK	QHDFFKSAMP	EGYVQERTIF
SCFP3A	TGKLPVPWPT	LVTTLTWGVQ	CFARYPDHMK	QHDFFKSAMP	EGYVQERTIF
mJuniper	TGKLPVPWPT	LVTTLT**W**GVQ	CF**A**RYPDHMK	QHDFFKSAMP	EGYVQERTIS
mYPet	TGKLPVPWPT	LVTTLGYGVQ	CFARYPDHMK	QHDFFKSAMP	EGYVQERTIF
SYFP2	TGKLPVPWPT	LVTTLGYGVQ	CFARYPDHMK	QHDFFKSAMP	EGYVQERTIF
mLemon	TGKLPVPWPT	LVT**S**L**G**YGVQ	CF**A**RYPDHMK	QHDFFKSAMP	EGYVQERTIS

	100	110	120	130	140
avGFP	FKDDGNYKTR	AEVKFEGDTL	VNRIELKGID	FKEDGNILGH	KLEYNYNSHN
mGreenlantern	FKDDGTYKTR	AEVKFEGDTL	VNRIVLKGID	FKEDGNILGH	KLEYNFNSHK
sfGFP	FKDDGTYKTR	AEVKFEGDTL	VNRIELKGID	FKEDGNILGH	KLEYNFNSHN
mChatreuse	FKDDGTYKTR	AEVKFEGDTL	VNRIELKG**SG**	FKEDGNILGH	KLEYN**Y**NSH**K**
mTurquoise2	FKDDGNYKTR	AEVKFEGDTL	VNRIELKGID	FKEDGNILGH	KLEYNYFSDN
SCFP3A	FKDDGNYKTR	AEVKFEGDTL	VNRIELKGID	FKEDGNILGH	KLEYNYISDN
mJuniper	FKDDGTYKTR	AEVKFEGDTL	VNRIELKG**SG**	FKEDGNILGH	KLEYN**YF**S**DK**
mYPet	FKDDGNYKTR	AEVKFEGDTL	VNRIELKGID	FKEDGNILGH	KLEYNYNSHN
SYFP2	FKDDGNYKTR	AEVKFEGDTL	VNRIELKGID	FKEDGNILGH	KLEYNYNSHN
mLemon	FKDDGTYKTR	AEVKFEGDTL	VNRIELKG**SG**	FKEDGNILGH	KLEYN**Y**NSH**K**

	150	160	170	180	190
avGFP	VYIMADKQKN	GIKVNFKIRH	NIEDGSVQLA	DHYQQNTPIG	DGPVLLPDNH
mGreenlantern	VYITADKQKN	GIKANFKTRH	NVEDGGVQLA	DHYQQNTPIG	DGPVLLPDNH
sfGFP	VYITADKQKN	GIKANFKIRH	NVEDGSVQLA	DHYQQNTPIG	DGPVLLPDNH
mChatreuse	VYITADKQKN	GIKANFKIRH	NVEDGSVQLA	DHYQQNTPIG	DGPVLLPDNH
mTurquoise2	VYITADKQKN	GIKANFKIRH	NIEDGGVQLA	DHYQQNTPIG	DGPVLLPDNH
SCFP3A	VYITADKQKN	GIKANFKIRH	NIEDGGVQLA	DHYQQNTPIG	DGPVLLPDNH
mJuniper	VYITADKQKN	GIKANFKIRH	NVEDGSVQLA	DHYQQNTPIG	DGPVLLPDNH
mYPet	VYITADKQKN	GIKANFKIRH	NIEDGGVQLA	DHYQQNTPIG	DGPVLLPDNH
SYFP2	VYITADKQKN	GIKANFKIRH	NIEDGGVQLA	DHYQQNTPIG	DGPVLLPDNH
mLemon	VYITADKQKN	GIKANFKIRH	NVEDGSVQLA	DHYQQNTPIG	DGPVLLPDNH

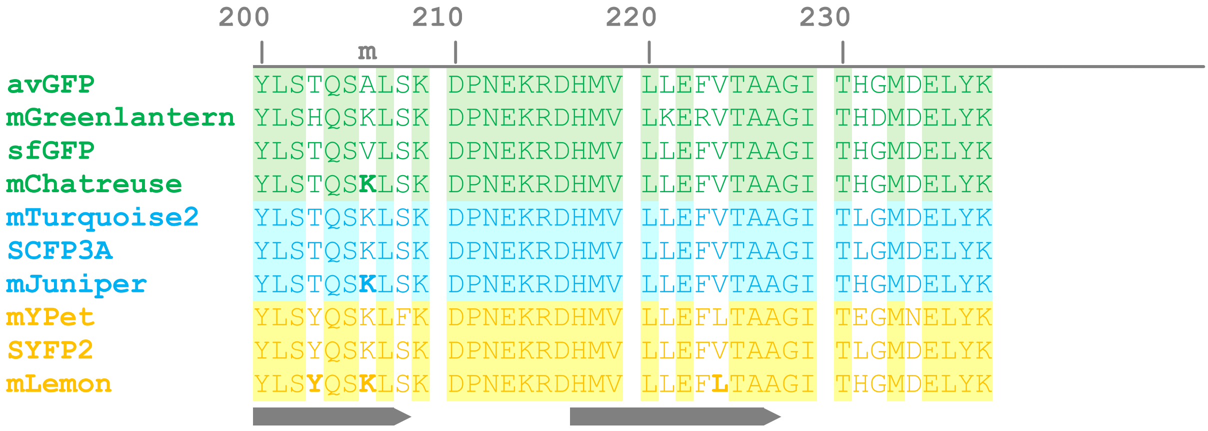

Figure S1: Alignments of avGFP derivatives used in this study. Numbering is shown relative to avGFP. τ : alpha helixes ; ➡ : beta sheets ; * : chromophore residues ; m : position of the monomerizing substitution (206K) (Zacharias et al., 2002). Substitutions introduced in the scope of this study are shown in bold. Note that our sfGFP variant does not contain the Q80R substitution as originally described and that our mYPet variant does not contain the valine insertion between positions 1 and 2. Also note that mNeongreen is not shown here due to low similarity with avGFP and derivatives.

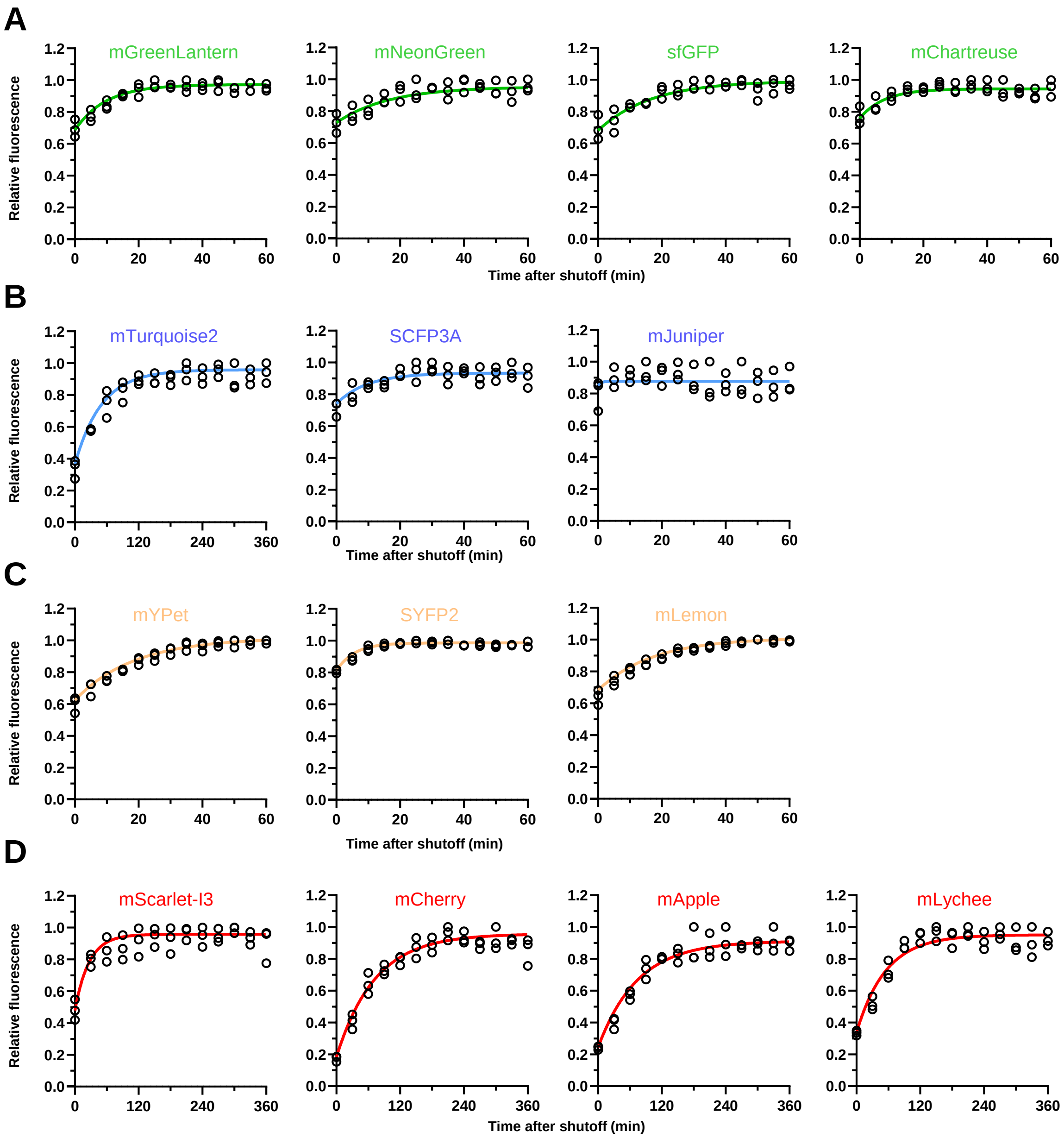

Figure S2: Maturation curves for green (A), cyan (B), yellow (C) and red (D) fluorescent proteins. Exponentially growing cultures of FP-producing bacteria were treated with 100 µg/mL erythromycin, 10 µg/mL tetracycline hydrochloride and 10 µg/mL rifampicin to arrest protein synthesis and enable dark fluorophores to fully mature. Fluorescence was read in 24-well plates every 5 min or 30 min under heating (37°C) and intermittent shaking. Data shows the mean and standard deviation of three independent replicates.

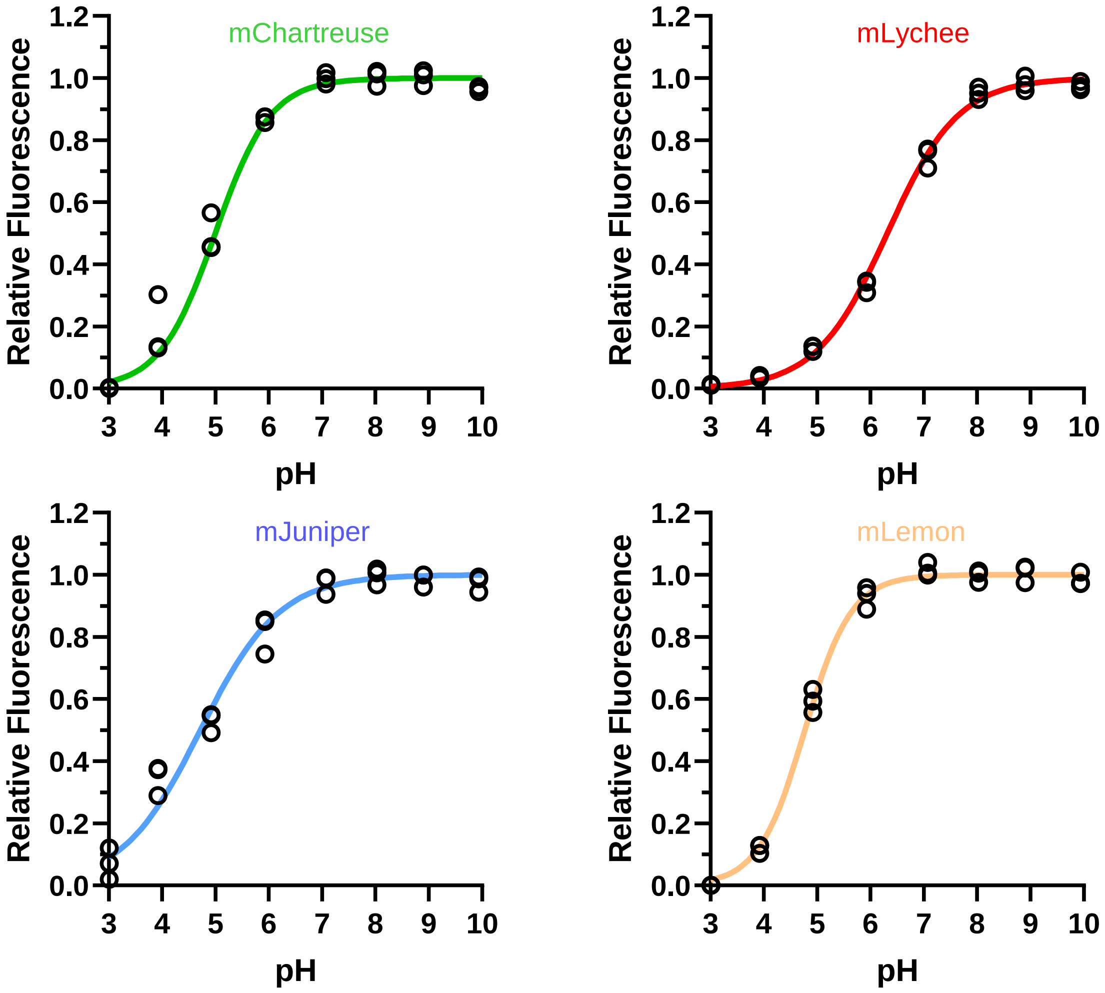

Figure S3: Effect of pH on fluorescence intensity. Purified mChartreuse, mJuniper, mLemon and mLychee in 10 mM Tris-HCl pH 8.0 were diluted 2x in citrate-phosphate-borate buffers at indicated pH. Data shows the mean and standard deviation of three independent replicates with a fitted Hill function for each triplicate.

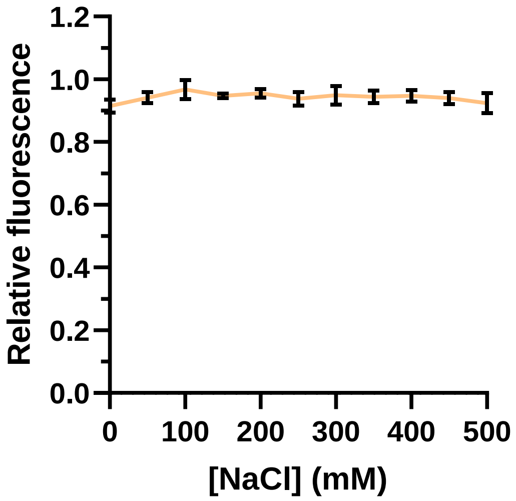

Figure S4: Effect of sodium chloride on mLemon fluorescence intensity. Purified mLemon in 10 mM Tris-HCl pH 8.0 was diluted in the same buffer containing increasing amounts of NaCl, with final concentrations shown on the x axis. Data shows the mean and standard deviation of three independent replicates.

	10	20	30	40
**DsRed**	M-----RSSK	NVIKEFMRFK	VRMEGTVNGH	EFEIEGEGEG	RPYEGHNTVK
**mCherry**	MVSKGEEDNM	AIIKEFMRFK	VHMEGSVNGH	EFEIEGEGEG	RPYEGTQTAK
**mScarlet-I3**	M-----DSTE	AVIKEFMRFK	VHMEGSMNGH	EFEIEGEGEG	RPYEGTQTAK
**mApple**	MVSKGEENNM	AIIKEFMRFK	VHMEGSVNGH	EFEIEGEGEG	RPYEAFQTAK
**mLychee**	M-----**DSTE**	AIIKEFMRFK	VHMEGSVNGH	EFEIEGEGEG	RPYEAFQTAK

	50	60	*** 70	80	90
**DsRed**	LKVTKGGPLP	FAWDILSPQF	QYGSKVYVKH	PADIPDYKKL	SFPEGFKWER
**mCherry**	LKVTKGGPLP	FAWDILSPQF	MYGSKAYVKH	PADIPDYLKL	SFPEGFKWER
**mScarlet-I3**	LKVTKGGPLP	FSWDILSPQF	MYGSRAFIKH	PADIPDYWKQ	SFPEGFKWER
**mApple**	LKVTKGGPLP	FAWDILSPQF	MYGSKVYIKH	PADIPDYFKL	SFPEGFRWER
**mLychee**	LKVTKGGPLP	FAWDILSPQF	MYGSK**A**YIKH	PADIPDYFK**Q**	SFPEGFRWER

	100	110	120	130 m	140
**DsRed**	VMNFEDGGVV	TVTQDSSLQD	GCFIYKVKFI	GVNFPSDGPV	MQKKTMGWEA
**mCherry**	VMNFEDGGVV	TVTQDSSLQD	GEFIYKVKLR	GTNFPSDGPV	MQKKTMGWEA
**mScarlet-I3**	VMIFEDGGTV	SVTQDTSLED	GTLIYKVKLR	GGNFPPDGPV	MQKRTMGWEA
**mApple**	VMNFEDGGII	HVNQDSSLQD	GVFIYKVKLR	GTNFPSDGPV	MQKKTMGWEA
**mLychee**	VMNFEDGGII	HVNQDSSLQD	GVFIYKVKLR	GTNFP**P**DGPV	MQK**R**TMGWE**P**

	150	160	170	180	190
**DsRed**	STERLYPRDG	VLKGEIHKAL	KLKDGGHYLV	EFKSIYMAKK	PVQLPGYYYV
**mCherry**	SSERMYPEDG	ALKGEIKQRL	KLKDGGHYDA	EVKTTYKAKK	PVQLPGAYNV
**mScarlet-I3**	STERLYPEDV	VLKGDIKMAL	RLKDGGRYLA	DFKTTYKAKK	PVQMPGAFNI
**mApple**	SEERMYPEDG	ALKSEIKKRL	KLKDGGHYAA	EVKTTYKAKK	PVQLPGAYIV
**mLychee**	SEERMYPEDG	ALKSEIKKRL	KLKDGGHYAA	EVKTTYKAKK	PVQLPGAYIV

	200	210	220	230
**DsRed**	DSKLDITSHN	EDYTIVEQYE	RTEGRHHLFL	
**mCherry**	NIKLDITSHN	EDYTIVEQYE	RAEGRHST--	GGMDE-LYK
**mScarlet-I3**	DRKLDITSHN	EDYTVVEQYE	RSVARHST--	GGSGGS
**mApple**	DIKLDIVSHN	EDYTIVEQYE	RAEGRHST--	GGMDE-LYK
**mLychee**	DIKLDIVSHN	EDYT**V**VEQYE	RAEGRHS**GSQ**	**GGSGGS**LYK

Figure S5: Alignments of DsRed derivatives used in this study. Numbering is shown relative to wild-type DsRed. ɤ: alpha helixes ; ➨: beta sheets ; *: chromophore residues ; m: position of the monomerizing substitution (S131P) identified in this study. Substitutions introduced in the scope of this study are shown in bold.

Table S1: Properties and source of plasmids and strains used in this study.

Plasmid	Properties	Reference
pNF02-mSc-I	Mini-F *cat proDp-mscarlet-I*	Goormaghtigh *et al.* (24)
pNF02-sfGFP	Mini-F *cat proDp-sfgfp*	This study
pNF02-mAppleNC	Mini-F *cat proDp-mapplenc*	This study
pNF02-mChartreuse	Mini-F *cat proDp-mchartreuse*	This study
pNF02-mJuniper	Mini-F *cat proDp-mjuniper*	This study
pNF02-mLemon	Mini-F *cat proDp-mlemon*	This study
pNF02-mLychee	Mini-F *cat proDp-mlychee*	This study
pDress-mTq2-link-mSc-I3	Source for mTurquoise2 & mScarlet-I3	Gadella *et al.* (20)
pR6K-sfGFP	Source for sfGFP	Lab collection
pNF02-mNG	Source for mNeongreen	Rousseau *et al.* (44)
pML31	Source for mYPet	Lab collection, R. Reyes-Lamothe
pDx-mSc3-SYFP2	Source for SYFP2	Gadella *et al.* (20)
pROD62	Source for mCherry	Lab collection, R. Reyes-Lamothe
mApple-pBAD	Source for mApple	Shaner *et al.* (13)
pFN01-mCh-sfGFP	Mini-F *cat proDp-mcherry-link-sfgfp*	This study
pFN01-mCh-mNG	Mini-F *cat proDp-mcherry-link-mneongreen*	This study
pFN01-mCh-mGL	Mini-F *cat proDp-mcherry-link-mgreenlantern*	This study
pFN01-mCh-mChartreuse	Mini-F *cat proDp-mcherry-link-mchartreuse*	This study
pFN01-mCh-mTq2	Mini-F *cat proDp-mcherry-link-mturquoise2*	This study
pFN01-mCh-SCFP3A	Mini-F *cat proDp-mcherry-link-scfp3a*	This study
pFN01-mCh-mJuniper	Mini-F *cat proDp-mcherry-link-mjuniper*	This study
pFN01-mTq2-mYPet	Mini-F *cat proDp-mturquoise2-link-mypet*	This study
pFN01-mTq2-SYFP2	Mini-F *cat proDp-mturquoise2-link-syfp2*	This study
pFN01-mTq2-mLemon	Mini-F *cat proDp-mturquoise2-link-mlemon*	This study
pFN01-mTq2-mCherry	Mini-F *cat proDp-mturquoise2-link-mcherry*	This study
pFN01-mTq2-mSc-I3	Mini-F *cat proDp-mturquoise2-link-mscarlet-i3*	This study
pFN01-mTq2-mApple	Mini-F *cat proDp-mturquoise2-link-mapple*	This study
pFN01-mTq2-mLychee	Mini-F *cat proDp-mturquoise2-link-mlychee*	This study
pUA66	ori_{pSC101} *aphA gfpmut2*	Zaslaver *et al.* (45)

pCLP	*ori*$_{pSC101}$ *aphA P*$_{clpXP}$*-clpP*	This study
pCLP-sfGFP	*ori*$_{pSC101}$ *aphA P*$_{clpXP}$*-clpP-sfgfp*	This study
pCLP-mNG	*ori*$_{pSC101}$ *aphA P*$_{clpXP}$*-clpP-mneongreen*	This study
pCLP-mGL	*ori*$_{pSC101}$ *aphA P*$_{clpXP}$*-clpP-mgreenlantern*	This study
pCLP-mChartreuse	*ori*$_{pSC101}$ *aphA P*$_{clpXP}$*-clpP-mchartreuse*	This study
pCLP-mTq2	*ori*$_{pSC101}$ *aphA P*$_{clpXP}$*-clpP-mturquoise2*	This study
pCLP-SCFP3A	*ori*$_{pSC101}$ *aphA P*$_{clpXP}$*-clpP-scfp3a*	This study
pCLP-mJuniper	*ori*$_{pSC101}$ *aphA P*$_{clpXP}$*-clpP-mjuniper*	This study
pCLP-mYPet	*ori*$_{pSC101}$ *aphA P*$_{clpXP}$*-clpP-mypet*	This study
pCLP-SYFP2	*ori*$_{pSC101}$ *aphA P*$_{clpXP}$*-clpP-syfp2*	This study
pCLP-mLemon	*ori*$_{pSC101}$ *aphA P*$_{clpXP}$*-clpP-mlemon*	This study
pCLP-mCherry	*ori*$_{pSC101}$ *aphA P*$_{clpXP}$*-clpP-mcherry*	This study
pCLP-mCherryS131P	*ori*$_{pSC101}$ *aphA P*$_{clpXP}$*-clpP-mcherry*$_{S131P}$	This study
pCLP-mSc-I3	*ori*$_{pSC101}$ *aphA P*$_{clpXP}$*-clpP-mscarlet-i3*	This study
pCLP-mApple	*ori*$_{pSC101}$ *aphA P*$_{clpXP}$*-clpP-mapple*	This study
pCLP-mAppleS131P	*ori*$_{pSC101}$ *aphA P*$_{clpXP}$*-clpP-mapple*$_{S131P}$	This study
pCLP-mLychee	*ori*$_{pSC101}$ *aphA P*$_{clpXP}$*-clpP-mlychee*	This study
pCP20	*ori*$_{pSC101(ts)}$ *bla cat cI857* P$_{L}$*-flp*	Lab collection
pET151	*ori*$_{pBR322}$ *bla lacI* P$_{T7lac}$	Life Technologies
pET151-mChartreuse	*ori*$_{pBR322}$ *bla lacI* P$_{T7lac}$*-mchartreuse*	This study
pET151-mJuniper	*ori*$_{pBR322}$ *bla lacI* P$_{T7lac}$*-mjuniper*	This study
pET151-mLemon	*ori*$_{pBR322}$ *bla lacI* P$_{T7lac}$*-mlemon*	This study
pET151-mLychee	*ori*$_{pBR322}$ *bla lacI* P$_{T7lac}$*-mlychee*	This study
pKNG101	*ori*$_{R6K}$ *strAB sacB oriT*$_{RP4}$	Kaniga *et al.* (46)
pKNG101-ftsZ-mCha	*ori*$_{R6K}$ *strAB sacB oriT*$_{RP4}$ *ftsZ-mchartreuse*$_{SW}$	This study
pKNG101-ftsZ-mNG	*ori*$_{R6K}$ *strAB sacB oriT*$_{RP4}$ *ftsZ-mneongreen*$_{SW}$	This study
pKNG101-mreB-mLy	*ori*$_{R6K}$ *strAB sacB oriT*$_{RP4}$ *mreB-mlychee*$_{SW}$	This study
pUA139-recA	*ori*$_{pSC101}$ *aphA P*$_{recA}$*-gfpmut2*	Zaslaver *et al.* (45)
pUArecA-mJuniper	*ori*$_{pSC101}$ *aphA P*$_{recA}$*-mjuniper*	This study
pUArecA-SCFP3A	*ori*$_{pSC101}$ *aphA P*$_{recA}$*-scfp3a*	This study
pSSB-mYPet	*ori*$_{pSC101}$ *bla ssb-mypet*	This study

pSSB-mLemon	*ori*$_{pSC101}$ *bla ssb-mlemon*	This study
**Strain**	**Genotype**	**Reference**
MG1655	Wild-type *Escherichia coli*	Lab collection
TB28	MG1655 Δ*lacIZYA::FRT*	Bernhardt & De Boer (42)
JW0427	BW25113 Δ*clpP::FRT-aphA-FRT*	Baba *et al.* (43)
LY3581	MG1655 Δ*clpP::FRT*	This study
BL21(DE3)	Protein production *E. coli* strain	Lab collection
FtsZ-mCha	TB28 *ftsZ-mchartreuse*$_{SW}$	This study
FtsZ-mNG	TB28 *ftsZ-mneongreen*$_{SW}$	This study
MreB-mChe	TB28 *mreB-mcherry*$_{SW}$ *yhdE::cat*	Bendezù *et al.* (38)
MreB-mLy	TB28 *mreB-lychee*$_{SW}$ *yhdE::cat*	This study

Table S2: Names and nucleotide sequences of primers used in this study.

Primer name	Primer sequence
bbpNF02 F	TAAGTGCACCTCTAGTATCACAC
bbpNF02 R	CATGCTAGCTTTCTCCTCTTTC
sfGFP02 F	GAAAGAGGAGAAAGCTAGCATGTCTAAAGGTGAAGAACTGTTC
sfGFP02 R	GTGATACTAGAGGTGCACTTATTTGTAGAGCTCATCCATGCCG
IpFN01 F	GGAGAAAGAAAAATGAAAACAGTGAGCAAGGGCGAGGAGC
IpFN01 R	CTAGAGGTGCACTTACTTGTACAAGGAGCC
VpFN01 F	CAAGTAAGTGCACCTCTAGTATCACAC
VpFN01 R	CACTGTTTTCATTTTTCTTTCTCCTCTTTCTCTAGTAAAAG
01misc F	CCGGTCGCCACCATGGTGAGCAAGGGCGAGGAG
01misc R	GTGTGATACTAGAGGTGCACTTACTTGTACAGCTCGTCCATGCC
01YPet F	CCGGTCGCCACCATGTCTAAAGGTGAAGAATTATTCAC
01YPet R	GTGTGATACTAGAGGTGCACTTATTTGTACAATTCATTCATACCCTC
01sfGFP F	CCGGTCGCCACCATGTCTAAAGGTGAAGAACTG
01sfGFP R	GTGTGATACTAGAGGTGCAC
VmTq2mCh F	GGCATGGACGAGCTCTACAAG
VmTq2mCh R	GCTCCTCGCCCTTGCTCAC
ImTq2mCh F	GTGAGCAAGGGCGAGGAGC
ImTq2mCh R	ACTTGTAGAGCTCGTCCATGCC
01mNGb F	CCGGTCGCCACCATGGTTTCTAAAGGTGAAGAAGACAATATGGC
01mNGb R	GTGTGATACTAGAGGTGCACTTATTTGTACAGTTCATCCATGCCC
mGL F	GCTACCGGTCGCCACCATGGTTTCTAAAGGTGAAG
VSCFP3A F	CAACTACATTAGCGACAACGTC
VSCFP3A R	CGCCCCAGGTCAGGGTGGTCACG
ISCFP3A F	GACCACCCTGACCTGGGGCGTGC
ISCFP3A R	CGTTGTCGCTAATGTAGTTGTACTCC
clpP F	CCCCGCTAGCGCTAAATTCGCACAAAGGC
clpP R	CCCGGATCCGCCACCGCCTGAATTACGATGGGTCAGAATCG
insCLP F	GTAATTCAGGCGGTGGCGGGCTACCGGTCGCCACCATG
insCLP R	CACGAGGCCCTTTCGTCTTGTGATACTAGAGGTGCACTTA
pCLP F	AAGACGAAAGGGCCTCGTG
pCLP R	CCGCCACCGCCTGAATTACG
mFruitS131P F	CCGACGGCCCCGTAATGCAG
mFruitS131P R	GGGGGAAGTTGGTGCCGCGC
pET151 F	CACCGCTGAGCAATAACTAGC
pET151 R	CTTTCTTAAAGTTAAACAAAATTATTTCTAGAGGGG
151FP F	GAAATAATTTTGTTTAACTTTAAGAAAGAGGAGAAAGCTAGCATG
151FP R	GTTATTGCTCAGCGGTGTGATACTAGAGGTGCAC
A_ftsZgfp_bamHi_Fw	TTCTACTTATGGTACCCGGGGATCCCATGTCACAACGATATGGCG
A_ftsZgfp_bamHi_Rev	CTTTAGACATCTCGAGGGTGGATCCTCCAACCGCTGTTTTACGCAG
B_ftsZgfp_Fw	AGCGGTTGGAGGATCCACCCTCGAGATGTCTAAAGGTGAAGAACTG
B_ftsZgfp_Rev	GAATCGTCTGGGTGGATCCCTCGAGTTTGTAGAGCTCATCCATGC
C_ftsZgfp_Fw	GCTCTACAAACTCGAGGGATCCACCCAGACGATTCAAATCGGTAGC
C_ftsZgfp_BamHi_Rev	CCCCCTGCAGGTCGACGGATCCTGCTTACGCAGGAATGCTGG
A_pko_mreBLy_bamHi_Fw	GGGATCGCGGCCGCGGACCGGATCCGTTTAACCTGCCGTTTAATC
A_mreBSWmLy_Rev	CTGTTGAATCCATGGAAGAACCGCTGCCCGGATAAGCCGAACCGA
B_mreBSWmLy_Fw	GGCTTATCCGGGCAGCGGTTCTTCCATGGATTCAACAGAAGCAAT
B_mreBSWmLy_Rev	GGACTTCATCGCCCGGTGCGCCAGACTTGTACAAACTTCCGCCAG
C_mreBSWmLy_Fw	TTTGTACAAGTCTGGCGCACCGGGCGATGAAGTCCGTGAAATCGA
A_pko_mreBLy_bamHi_rev	GGATCGCGGCCGCTCTAGAGGATCCATATCGTTGCGCAGCACCTG
pUA66 fluoswap F	ACCTGCAGGCATGCAAGC
pUA66 fluoswap R	CATATGTATATCTCCTTCTTAAATCTAG
mJunUA66 F	GATTTAAGAAGGAGATATACATATGTCTAAAGGTGAAGAACTGTTC
mJunUA66 R	GCTTGCATGCCTGCAGGTTTATTTGTAGAGCTCATCCATGCC
SCFP3Aopt R	CTTGTGGCCGTTAACATCACC
SCFP3Aopt F	GATGTTAACGGCCACAAGTTCAGCG
CFPUA66 R	GCTTGCATGCCTGCAGGTTTACTTGTAGAGCTCGTCCATGCC
